# Hypertension as the Most Frequent Cardiovascular Cause of Readmissions After Heart Failure Hospitalization: A Nationwide Analysis

**DOI:** 10.7759/cureus.87244

**Published:** 2025-07-03

**Authors:** Kyle E Thurmann, Trisha G Mukherjee, Joseph G Dantin, Paul Kang, Michael D White

**Affiliations:** 1 School of Medicine, Creighton University School of Medicine, Phoenix, USA; 2 School of Medicine, Rocky Vista University College of Osteopathic Medicine, Parker, USA; 3 Cardiology, Valleywise Health Medical Center, Phoenix, USA

**Keywords:** cardiovascular readmission, healthcare utilization, heart failure, hospital readmission, hypertension, nationwide readmission database, transitional care

## Abstract

Background

Cardiovascular (CV) readmissions after heart failure (HF) hospitalization represent a significant source of morbidity and healthcare burden. Although hypertension (HTN) is a key precursor of HF, its impact on CV-specific readmissions after discharge is less well characterized.

Methods

We conducted a retrospective cohort study using the Nationwide Readmissions Database, spanning 2016 to 2022. Adult patients hospitalized with a primary diagnosis of HF were included. CV-specific readmissions within 30 days and one year were identified using ICD-10 codes for HTN, HF, myocardial infarction, arrhythmias, stroke, and thromboembolic conditions. Demographic, clinical, and hospital-level variables were evaluated to identify factors associated with readmission risk.

Results

Among 31,886,859 weighted hospitalizations for HF, HTN, or HTN crisis, HTN crisis was the most frequent cause of CV-specific readmissions, comprising 64.80% (N=20,662,685) of 30-day and 65.10% (N=20,758,345) of one-year events. All comparisons vs. HTN were highly significant (p<0.001) with large effect sizes (phi-coefficients=0.53-0.69).

Conclusion

HTN is the most frequent diagnosis leading to CV-specific readmissions after HF discharge. These findings underscore the need for prioritized BP management to prevent recurrent admissions and improve outcomes.

## Introduction

Cardiovascular (CV) readmissions pose significant clinical and economic burdens, particularly among patients with heart failure (HF). Identifying specific diagnostic contributors to these readmissions can optimize preventive strategies and improve patient outcomes. As of 2012, the total cost of HF in the United States was estimated at $30.7 billion, with projections indicating a 127% increase to $69.8 billion by 2030, or approximately $244 per adult in the United States [1]. A systematic review from 2014 to 2020 found a median annual cost of $24,383 per patient, with hospitalizations accounting for the largest share at $15,879 [2]. In addition to direct costs, recurrent hospitalizations exacerbate the burden of HF and further strain healthcare systems. An analysis of over 2.6 million HF admissions in the United States from 2010 to 2014 revealed that readmissions, along with comorbidities and invasive procedures, are major cost drivers [3]. 

Hypertension (HTN), coronary heart disease, smoking, obesity, and diabetes collectively account for over half of new HF cases. HTN alone accounts for 20% of incident HF cases, with a greater impact in females (28%) compared to males (13%) [4]. The Framingham Heart Study reported that 91% of individuals who developed HF had a prior history of HTN over the preceding two decades [5,6]. Additional data show that HTN doubles the risk of HF in men and triples it in women, contributing to 39% and 59% of HF cases, respectively [7]. Despite therapeutic advancements, outcomes following HF hospitalization remain suboptimal. In a cohort of 2,494 patients discharged between 2015 and 2016, 49.1% were rehospitalized within six months, 47.4% of those for HF itself, while 34.1% presented to the ED and 15.5% died during that period [8]. 

Most national evaluations currently emphasize all-cause readmission, which obscures the specific CV diagnoses driving rehospitalizations. This diagnostic ambiguity hinders the development of targeted, risk-specific interventions and limits optimal resource allocation. Understanding which CV conditions most often lead to readmission is vital for informing outpatient care pathways and enhancing transitional care planning.

Given the focus on all-cause readmissions in prior studies and the lack of diagnosis-specific analyses of CV readmissions, our objective was to determine which CV diagnosis represents the leading contributor to readmissions following HF hospitalization at both 30 days and one year. A prior Medicare analysis revealed that 30-day readmissions after HF hospitalization are frequent and span a wide range of diagnoses, with more than 60% occurring within the first 15 days and no substantial variation by age, sex, or race [9]. However, prior research has not identified the specific CV drivers of readmission, underscoring the need for diagnosis-level insights to guide targeted interventions.

## Materials and methods

Data source and study design

We conducted a retrospective cohort study utilizing the Nationwide Readmissions Database (NRD), a publicly available dataset developed as part of the Healthcare Cost and Utilization Project (HCUP) by the Agency for Healthcare Research and Quality. The NRD comprises de-identified discharge data from 30 states, spanning the years 2016 to 2022, and represents approximately 60.0% of all inpatient hospitalizations and 61.1% of the national resident population [10]. 

Study population and inclusion criteria

We included adult patients aged 18 years or older who were hospitalized with a primary diagnosis of HF, identified using ICD-10-CM codes I50.1 through I50.9. To maintain a consistent analytic cohort, we excluded patients who died during the index hospitalization, were discharged against medical advice, were transferred to another acute care facility, or had a planned readmission. Records missing essential demographic or outcome data were also excluded. The same cohort used in this study was also analyzed in a separate investigation focused on psychiatric comorbidities and their association with CV-specific readmissions following HF hospitalization. That study examined different exposures and outcomes and was submitted as an independent manuscript.

Outcome definition: CV-specific readmissions

The primary outcome was a CV-specific readmission within 30 days or one year following discharge. CV-specific readmissions were identified using ICD-10 codes for the following diagnostic categories: HF and pulmonary edema (I50.1-I50.9, J81.0-J81.1), acute myocardial infarction (MI) (I21.0-I21.9), arrhythmias and conduction disorders (I47.0-I49.9), stroke and transient ischemic attack (I60.0-I63.9, G45.9), HTN and HTN crisis (I10-I16), pulmonary circulation disorders (I26.0-I27.9), and venous thromboembolism (I82.0-I82.9). These categories are consistent with established groupings in CV health services research.

Covariates and patient characteristics

Patient-level demographic variables included age, sex, income quartile (based on ZIP code), and primary payer. Hospital-level factors included location (urban vs. rural), teaching status, and bed size. Additional covariates included admission day (weekday vs. weekend), discharge disposition (home, skilled nursing facility, home healthcare, or other institutional care), and comorbidity burden, measured using the Charlson Comorbidity Index (CCI), a validated metric for predicting mortality and healthcare utilization [11].

Statistical analysis

Categorical variables were compared using survey-weighted chi-square (χ²) tests of independence, applying HCUP discharge-level weights to generate national estimates. Analyses were stratified by 30-day and one-year CV-specific readmission status. We report weighted summary statistics (proportions ± standard error) for demographic, clinical, and hospital characteristics. For each diagnosis vs. HTH or HTN crisis, we present χ² (degrees of freedom=1), two-sided p-values, and phi-coefficients (φ) to quantify the association strength, alongside relative risks from weighted proportions. A two-sided p<0.001 was considered statistically significant.

## Results

Our analysis comprised 31,886,859 weighted HF hospitalizations from the NRD (2016-2022). The cohort had a mean age of 71.3 years, with females comprising 48.9% (N=15,592,674) of the admissions. Most patients were insured through Medicare (75.7%, N=24,138,352) and resided in urban areas (82.4%, N=26,274,772), with 32.8% (N=10,458,890) falling within the lowest income quartile. Nearly one in four hospitalizations occurred on weekends (24.4%, N=7,780,394). Large hospitals (53.8%, N=17,155,130) and urban teaching centers (69.8%, N=22,257,028) accounted for the majority of care. Over 74% (N=23,883,257) of patients had a CCI score ≥3. Discharge locations were evenly divided among home (45.6%, N=14,540,408), home health services (25.8%, N=8,226,810), and designated center (25.7%, N=8,194,923). Baseline characteristics are presented in Table 1.

**Table 1:** Demographic, clinical, and hospital characteristics of the study population This table presents weighted baseline characteristics of hospitalizations for heart failure included in the Nationwide Readmissions Database (N=31,886,859). Variables include patient demographics (age, sex, and income quartile), primary payer type, admission setting (weekend vs. weekday), hospital location and teaching status, hospital bed size, Charlson Comorbidity Index, and discharge disposition. All values are expressed as weighted percentages with standard errors. Income quartiles reflect income classification based on ZIP code. N, weighted frequency; SE, standard error

Demographics (weighted)	Overall (n=31,886,859)	
	% (N)	SE
Age, years	Mean = 71.3	0.004
Sex, female	48.9 (15,592,674)	0.013
Income quartile		
1	32.8 (10,458,890)	0.011
2	27.7 (8,832,660)	0.011
3	22.9 (7,302,091)	0.011
4	16.7 (5,325,105)	0.009
Primary payer		
Medicare	75.7 (24,138,352)	0.011
Medicaid	10.1 (3,220,573)	0.007
Private	10.0 (3,188,686)	0.008
Self-pay	1.79 (570,775)	0.003
Other	2.4 (765,285)	0.004
Weekend admission	24.4 (7,780,394)	0.011
Patient location		
Urban	82.4 (26,274,772)	0.008
Rural	17.6 (5,612,087)	0.008
Hospital location		
Urban/non-teaching	20.4 (6,504,919)	0.002
Urban/teaching	69.8 (22,257,028)	0.003
Rural	9.8 (3,124,912)	0.002
Hospital bed size		
Small	18.5 (5,899,069)	0.002
Medium	27.7 (8,832,660)	0.002
Large	53.8 (17,155,130)	0.003
Charlson Comorbidity Index		
0–1	7.3 (2,327,741)	0.007
2	17.8 (5,675,861)	0.009
>3	74.9 (23,883,257)	0.011
Disposition at discharge		
Home	45.6 (14,540,408)	0.012
Short-term facility	1.2 (382,642)	0.003
Designated center	25.7 (8,194,923)	0.011
Home healthcare	25.8 (8,226,810)	0.011
Against medical advice	1.7 (542,077)	0.003
Unknown	0.047 (14,987)	6.5e-6

**Figure 1:**
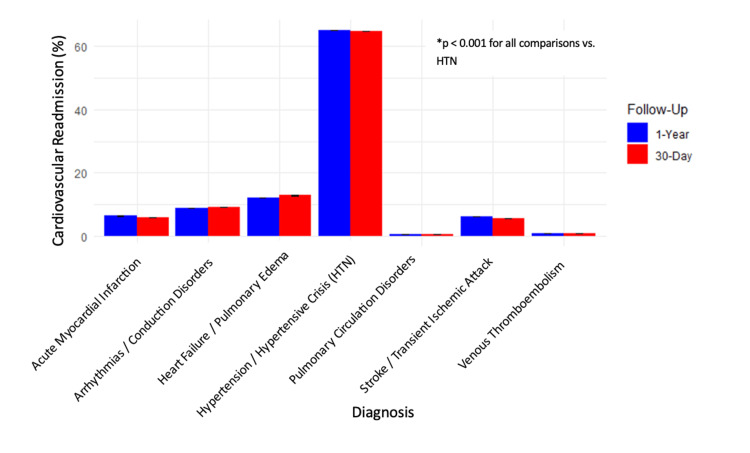
Comparison of 30-day and one-year cardiovascular-specific readmissions by primary diagnosis following heart failure hospitalization This figure illustrates the percentage of cardiovascular-specific readmissions attributed to each diagnostic category at 30 days and one year following an index heart failure hospitalization, based on weighted national estimates (N=31,886,859). The most frequent cause of readmission at both time points was hypertension or hypertensive crisis, accounting for 64.80% (N=20,662,685) at 30 days and 65.10% (N=20,758,345) at one year. *All comparisons vs. hypertension or hypertensive crisis were statistically significant (p<0.001). HTN, hypertension

Among CV-specific readmissions, HTN or HTN crisis was the most frequent diagnostic category at both 30-day and one-year intervals, comprising 64.80% (N=20,662,685) and 65.10% (N=20,758,345) of cases. HF or pulmonary edema was the second most frequent diagnosis, accounting for 12.90% (N=4,113,504) and 12.10% (N=3,858,310) of readmissions. This was followed by arrhythmias or conduction disorders at 9.2% (N=2,946,346) and 8.8% (N=2,802,855), and acute MI at 5.90% (N=1,881,325) and 6.37% (N=2,031,193). Less frequent causes included stroke or transient ischemic attack (5.6%, N=1,788,853 and 6.1%, N=1,957,853), venous thromboembolism (0.8%, N=267,850 and 0.8%, N=251,906), and pulmonary circulation disorders (0.6%, N=197,699 and 0.6%, N=194,510), with values listed for 30-day and one-year intervals, respectively. All comparisons vs. HTN or HTN crisis were statistically significant (p<0.001), with φ-coefficients ranging from 0.53 to 0.69. Diagnosis-specific readmission patterns are detailed quantitatively in Table 2 and visually presented in Figure 1.

**Table 2:** Weighted distribution of 30-day and one-year cardiovascular-specific readmissions by primary diagnosis following heart failure hospitalization Data are presented as weighted percentage ± standard error. Between-group comparisons vs. hypertension or hypertensive crisis were made using survey-weighted chi-square tests (degrees of freedom = 1), with two-sided p-values and phi-coefficients to quantify association strength. All p<0.001. A phi-coefficient of ~0.10 means a slight difference in readmission risk for hypertension vs. other conditions, ~0.30 a moderate difference, and ≥0.50 a large difference. Our results indicate that hypertension or hypertensive crisis patients are substantially more likely to be readmitted than patients with other diagnoses. df, degrees of freedom; N, weighted frequency; SE, standard error; χ², chi-square test of independence; φ, phi

N=31,886,859	30-day readmission					One-year readmission				
	% (N)	SE	χ²(df=1) vs. HTN	p-values vs. HTN	φ coefficient vs. HTN	% (N)	SE	χ²(df=1) vs. HTN	p-values vs. HTN	φ-coefficient vs. HTN
Acute myocardial infarction	5.90 (1,881,325)	0.024	24,202,174	<0.001	0.62	6.37 (2,031,193)	0.017	23,946,025	<0.001	0.61
Arrhythmias/conduction	9.24 (2,946,346)	0.030	21,108,975	<0.001	0.58	8.79 (2,802,855)	0.020	21,700,892	<0.001	0.58
Heart failure/pulmonary edema	12.90 (4,113,504)	0.035	18,076,867	<0.001	0.53	12.10 (3,858,310)	0.023	18,896,344	<0.001	0.54
Hypertension/hypertensive crisis	64.80 (20,662,685)	0.049				65.10 (20,758,345)	0.034			
Pulmonary circulation	0.62 (197,699)	0.008	29,836,652	<0.001	0.68	0.61 (194,510)	0.006	30,057,395	<0.001	0.69
Stroke/transient ischemic attack	5.61 (1,788,853)	0.024	24,486,831	<0.001	0.62	6.14 (1,957,853)	0.017	24,168,617	<0.001	0.62
Venous thromboembolism	0.84 (267,850)	0.009	29,581,492	<0.001	0.68	0.79 (251,906)	0.006	29,848,196	<0.001	0.68

Patients readmitted for HTN or HTN crisis were over five times more likely to be rehospitalized than those with HF or pulmonary edema (relative risk or RR=5.02 at 30 days; 5.38 at one year), over seven times more likely than those with arrhythmias or conduction disorders (RR=7.01; 7.41), and over 10 times more likely than those with acute MI (RR=10.98; 10.22). Relative risks were even higher when compared to less frequent diagnoses such as stroke or transient ischemic attack (RR=11.55; 10.60), venous thromboembolism (RR=77.14; 82.41), and pulmonary circulation disorders (RR=104.52; 106.72). Values are listed for 30-day and one-year intervals, respectively. All diagnosis-specific differences in relative readmission risk were statistically significant based on weighted chi-squared tests (p<0.001). Relative risk values for each diagnosis compared to HTN or HTN crisis at both time points are summarized in Table 3.

**Table 3:** Relative risk of cardiovascular-specific readmission for each diagnosis compared to the hypertension or hypertension crisis diagnostic category This table displays the relative risk of 30-day and one-year cardiovascular-specific readmissions for various diagnostic categories using hypertension or hypertensive crisis as the reference group. Relative risk values greater than one indicate a lower likelihood of readmission compared to hypertension or hypertensive crisis. All comparisons are based on weighted proportions from the Nationwide Readmissions Database. Relative risks were calculated using weighted proportions, not regression-based models. N, weighted frequency

Diagnosis (N=31,886,859)	Relative risk at 30 days	Relative risk at one year
Acute myocardial infarction	10.98	10.22
Arrhythmias/conduction disorders	7.01	7.41
Heart failure/pulmonary edema	5.02	5.38
Pulmonary circulation disorders	104.52	106.72
Stroke/transient ischemic attack	11.55	10.60
Venous thromboembolism	77.14	82.41

## Discussion

This study identified HTN or HTN crisis as the most frequent cause of CV-specific readmissions at both 30 days and one year following hospitalization for HF. All comparisons vs. HTN or HTN crisis demonstrated large effect sizes (φ = 0.53-0.69), underscoring the outsized role of HTN in post-discharge CV outcomes and suggesting that intensified blood pressure (BP) management may be a key opportunity for intervention. Diagnostic patterns were consistent across time intervals, with recurrent HF, arrhythmias, and MI also contributing substantially to rehospitalizations.

Our results extend prior evidence that HTN not only contributes to HF development but also disproportionately drives post-discharge CV readmissions [4,6,12-15]. These findings underscore the critical importance of BP control for both HF prevention and the reduction of recurrent CV hospitalizations. Advances in BP management have been shown to significantly lower CV event risk, with meta-analyses demonstrating that a 10 mmHg systolic BP reduction decreases HF risk by 28% and improves CV mortality across antihypertensive classes [16-20].

Despite advances in BP management and evolving strategies in transitional and outpatient HF care, our data suggest that HTN remains a prevalent, inadequately managed, and perhaps underappreciated contributor to CV-specific readmissions after HF hospitalization. Possible explanations for this unexpected finding include suboptimal BP control at discharge, incomplete initiation or titration of antihypertensive medications, gaps in patient education on salt restriction and medication adherence, and low thresholds for hospitalization for HTN in certain healthcare settings. Interventions such as remote BP monitoring, medication adherence support, and timely outpatient follow-up are crucial to reducing readmission risk [21,22]. Programs such as the Risk-HF trial, which combined risk-guided disease management with handheld ultrasound and AI-based self-care tools, illustrate the feasibility and potential of diagnosis-specific transitional care models to improve outcomes [23]. National-level strategies such as pre-discharge follow-up scheduling, medication reconciliation, and direct communication with outpatient providers have similarly shown benefits in lowering 30-day HF readmission rates [24].

Our findings also align with global HTN trends. A systematic analysis across 90 countries reported that 1.4 billion adults had HTN in 2010, with over 1 billion residing in low- and middle-income countries. Notably, these regions experienced a 7.7% increase in prevalence and minimal improvement in control rates over the prior decade. These widening disparities highlight both the growing burden and the persistent gaps in HTN care. For patients recently hospitalized with HF, such systemic shortcomings may exacerbate the risk of preventable CV readmissions, particularly in settings with limited access to consistent outpatient care [25].

Future efforts should evaluate the effectiveness of diagnosis-specific strategies, such as HTN-targeted management, within structured outpatient programs like those in the Risk-HF trial and assess their impact on long-term HF outcomes. Approaches that combine risk stratification, handheld ultrasound-guided fluid monitoring, and AI-driven self-care education could strengthen early post-discharge care, lower rates of preventable CV readmissions, and improve overall prognosis in HF populations [23]. Additionally, it would be valuable to examine the presence and impact of coexisting conditions that may complicate BP management, such as diabetes, obesity, renal dysfunction, left ventricular hypertrophy, or sleep apnea syndrome, and how these influence readmission risk and BP control after discharge.

Limitations of this study include reliance on administrative claims data, which may be affected by misclassification or coding errors. The use of ICD-10 codes limits insight into disease severity and medication adherence. In particular, HTN or HTN crisis readmissions were identified using ICD-10-CM discharge codes, as the NRD does not capture clinical thresholds (e.g., sustained BP >200/120 mmHg or refractory HTN) or outpatient data such as actual BP readings or medication titration, limiting our ability to assess the direct mechanisms behind these readmissions. Finally, the NRD lacks reliable stratification by HF phenotype, which may obscure subtype-specific trends in readmissions or treatment response. As a result, our analysis reflects aggregate patterns across HF populations rather than tailored insights by ejection fraction. Despite these limitations, the large sample size and diagnosis-specific focus provide clinically and policy-relevant insights that enhance the generalizability and applicability of the findings.

## Conclusions

This nationwide analysis shows that HTN or HTN crisis is the most frequent cause of CV-specific readmissions following hospitalization for HF. While prior research has established HTN as a key contributor to HF development, our findings extend this understanding by demonstrating its dominant role in recurrent CV admissions after discharge. By shifting the focus from broad readmission metrics to diagnosis-specific drivers such as HTN, clinicians and policymakers can better design targeted interventions that reduce preventable rehospitalizations, improve patient outcomes, and support value-based care. These results underscore the critical need to integrate aggressive BP control into post-HF management strategies, and future efforts should prioritize structured, post-discharge care models centered on HTN management to reduce the long-term burden of CV disease.
